# Tumor Budding: The Name is EMT. Partial EMT.

**DOI:** 10.3390/jcm5050051

**Published:** 2016-04-29

**Authors:** Alexandru Dan Grigore, Mohit Kumar Jolly, Dongya Jia, Mary C. Farach-Carson, Herbert Levine

**Affiliations:** 1 Departments of BioSciences, Rice University, Houston, TX 77005-1827, USA; Alexandru.Dan.Grigore@rice.edu; 2 Departments of Bioengineering, Rice University, Houston, TX 77005-1827, USA; mkjolly.15@gmail.com; 3 Departments of Physics and Astronomy, Rice University, Houston, TX 77005-1827, USA; 4 Center for Theoretical Biological Physics, Rice University, Houston, TX 77005-1827, USA; dyajia@gmail.com; 5 Graduate Program in Systems, Synthetic and Physical Biology, Rice University, Houston, TX 77005-1827, USA

**Keywords:** tumor budding, epithelial-mesenchymal transition, EMT, cancer

## Abstract

Tumor budding is a histological phenomenon encountered in various cancers, whereby individual malignant cells and/or small clusters of malignant cells are seen in the tumor stroma. Postulated to be mirror epithelial-mesenchymal transition, tumor budding has been associated with poor cancer outcomes. However, the vast heterogeneity in its exact definition, methodology of assessment, and patient stratification need to be resolved before it can be routinely used as a standardized prognostic feature. Here, we discuss the heterogeneity in defining and assessing tumor budding, its clinical significance across multiple cancer types, and its prospective implementation in clinical practice. Next, we review the emerging evidence about partial, rather than complete, epithelial-mesenchymal phenotype at the tumor bud level, and its connection with tumor proliferation, quiescence, and stemness. Finally, based on recent literature, indicating a co-expression of epithelial and mesenchymal markers in many tumor buds, we posit tumor budding to be a manifestation of this hybrid epithelial/mesenchymal phenotype displaying collective cell migration.

## 1. Tumor Budding: Introducing the Concept

In histological jargon, “tumor budding” basically means the presence of clusters of undifferentiated malignant cells in the tumor stroma, which are located mainly (but not exclusively) in close proximity ahead of the invasive front of a tumor (reviewed in [1,2,3]). The phenomenon was first described in the Japanese medical literature by Imai, in 1949 (reviewed in [4]), in relation to stomach cancer, followed by other Japanese authors, in the 1950–1960s, who found correlations with prognosis in cancers of the tongue, larynx, breast, stomach, colon, rectum, and cervix. Tumor budding was revisited after two decades in the late 1980s in relation to colorectal cancer (CRC) patients (reviewed in [5], also see [6]).

A phenomenon compatible with the definition of tumor budding was independently described in the mid-1980s by Gabbert and colleagues in experimentally-induced colon cancers in mice treated with dimethylhydrazine-dihydrochloride. The invasive front of the lesions displayed a striking disorganization at the tumor architecture level (*i.e.*, loss of glandular aspect for differentiated carcinomas and loss of trabecular aspect for undifferentiated carcinomas, respectively) along with a dedifferentiation at the cellular level. These changes ultimately resulted in isolated tumor cells showing a uniform phenotype, irrespective of the differentiation of the main tumor mass (*i.e.*, differentiated or undifferentiated carcinomas). The phenotype had features compatible with cell mobility (loss of cell junctions and basement membrane, presence of cytoplasmic microfilaments and pseudopodia, versatile cell shape), which is why the whole process at the invasive front was suggested to help mobilize the cancer cells from the main tumor mass, followed by invasion of host tissues through locomotion [7]. Similarly, in surgical samples from patients with colon cancer, poorly differentiated malignant cells were found moving out ahead of the invasive front, either individually or in clusters. Each of the clustered cells displayed one large cytoplasmic flap, similar to a leading edge, while single cells displayed cytoplasmic projections similar to lamellipodia. All these features are compatible with locomotion [8].

The definition raised some terminology-related critiques. It was suggested that the term “budding”, if to be used correctly, would imply that the clusters of neoplastic cells remain connected to the main tumor, which was not the case in the 2D histological sections studied [9]. However, this critique was properly addressed by Carr and colleagues [8] through serial sections of colon cancer, and then in more detail by Bronsert and colleagues [10] through 3D reconstructions from 2D serial sections of various tumor types. These studies clearly showed that tumor budding is not a static snapshot, but rather a dynamic process by which the tumor actually extends numerous fingerlike projections, each containing numerous cells, which, at a later point, break apart from the main tumor mass as small cell clusters. Such an extending bud, if seen in a 2D section, might give the false impression that a cell cluster has already detached from the main tumor mass. In a nutshell, 2D sections show only small cell clusters that appear to have lost connection with the main tumor mass, whereas serial sections and 3D reconstructions give the real picture of a cocktail of growing buds that remain contiguous with the tumor and bud tips that have truly detached.

We next move on to describing how tumor budding is defined, identified, and quantified in tumor tissue. Although budding is being studied in a growing variety of cancers, we hereby discuss the general principles of assessment by using CRC as a model. We considered this suitable because CRC was the first cancer type in which budding was addressed systematically, thus providing the most abundant and consistent body of literature, which accounts for the standardization efforts in this emerging subfield.

## 2. Tumor Budding in Colorectal Cancer: Concepts and Methodologies

Tumor budding is an additional prognostic factor for CRC according to the Union for International Cancer Control (UICC) [11], and a potential prognostic factor in early CRC according to the European Society for Medical Oncology consensus guidelines [12]. According to the latter, malignant polyps, which display tumor budding, should undergo standard resection [12]. However, tumor budding has not yet entered routine clinical assessment as there is no consensus regarding the exact definition, the methodology of assessment, and the best way it can be used to stratify patients and assign them to prognostic categories, followed by appropriate therapeutic decisions. Therefore, consensus pathological criteria for defining, identifying, and quantifying tumor budding, are necessary, based on which statistically robust clinical trials with multivariate analysis should be conducted to unequivocally confirm its prognostic value [11].

Considerable heterogeneity exists in the methodology used for assessing tumor budding. The main differences can be categorized as follows: (i) definition, including choice of cutoff values for cell number within a bud-looking structure; (ii) identification of bud-looking structures, namely staining method: hematoxylin and eosin (H&E) staining *vs.* pan-cytokeratin immunostaining; (iii) final identification of buds following exclusion of bud-looking structures that are not true buds; (iv) region(s) of interest: assessment across the whole tumor (*i.e.*, all blocks or slides) or in the region displaying maximal budding; (v) magnification (chosen by the observer) and field-of-view size (which can vary according to the particular instrument used); (vi) quantification of tumor budding intensity: subjective patterns (descriptive) *vs.* semiquantitative (relative size, *i.e.*, ratio between the width of tumor budding region and width of invasive front) *vs.* quantitative (bud counts); (vii) choice of prognostic cutoff values; and (viii) applicability of each method to the stage of the primary lesion. A great number of combinations and adaptations of these methodological ingredients can be found in the literature (excellently reviewed in [2,3,13]).

Starting from the general definition above, various ways can be used to categorize a histological structure as a tumor bud. One of the first studies published in Western literature by Morodomi and colleagues defined buds as isolated undifferentiated malignant cells or clusters of ≥5 malignant cells showing a microtubular structure [6], and some authors followed this definition ([14,15]; see review by Koelzer and colleagues [3] for more references). Ueno and colleagues defined buds as isolated malignant cells or foci of ≤4 clustered malignant cells in the stroma at the invasive front of the tumor [16]. While arbitrarily chosen, this cutoff value for defining a structure as a bud has been widely used in the literature since the original publication [13,17,18,19,20,21,22,23,24,25,26,27,28]. Some authors slightly drifted away from this and increased the cutoff value by one cell, thus defining buds as isolated malignant cells or foci of ≤5 clustered malignant cells [29,30,31]. Recent accounts tend to favor this latter cutoff definition value [3].

Following the chosen definition, tumor buds are sought for in the tissue sample. However, there are certain situations when it is particularly difficult to decide, in routine H&E staining, whether a bud-looking structure is actually a bud. These situations include: (i) abundant inflammatory infiltrate at the invasive front, which makes it hard to distinguish between real buds and activated lymphocytes and histiocytes; (ii) abundant stromal reaction at the invasive front, which makes it hard to distinguish between real buds and stromal cells; (iii) fragmentation of tumor glands induced by abundant inflammatory infiltrate, which gives them a bud-looking appearance (in fact tumor budding is inversely correlated with the intensity of inflammation [32]); (iv) retraction artifacts around fragmented tumor glands, which gives them a bud-looking appearance; and (v) fragments of tumor tissue surrounded by an abundant mucinous extracellular matrix, which gives them a bud-looking appearance. For (i) and (ii), where H&E staining cannot easily distinguish between real buds and other structures, a cytokeratin immunostaining should be used. For (iii)–(v), where tissue fragmentation gives the false impression of budding, the fragments should be excluded from scoring [33].

Five of the most cited methods in the literature are the following: (i) Hase *et al.*, 1993 [5] (assessment by subjective perception; region of interest spanning entire tumor; classification as none/mild *vs.* moderate/severe); (ii) Ueno *et al.*, 2002 [16] (assessment by bud counting; region of interest spanning microscope field displaying maximal budding; magnification 250×; field area = 0.385 mm^2^; classification as low grade (<10 buds) *vs.* high-grade (≥10 buds)); (iii) Ueno *et al.*, 2004 (assessment by bud counting; region of interest spanning one microscope high-power field (1 HPF) displaying maximal budding; magnification 250×, field area = 0.785 mm^2^; classification as negative (<5 buds) *vs.* positive (≥5 buds)); (iv) Nakamura *et al.*, 2005 (assessment by semiquantitative score, *i.e.*, percentage involved by tumor budding; region of interest spanning entire advancing margin; classification as low grade (none/mild, width of budding region <1/3 of the width of advancing margin) *vs.* high-grade (moderate, width of budding region = 1/3–2/3 of the width of advancing margin; marked, width of budding region >2/3 of the width of advancing margin); (v) conventional Wang [22] (assessment by bud counting; region of interest spanning 5 HPF displaying maximal budding (detected by a preliminary scanning at 40×) or 5 randomly selected HPF (if buds could not be observed by preliminary scanning at 40×); magnification 200×; field area = 0.94985 mm^2^; classification as high if median score ≥ 1 *vs.* low if median score = 0); and (vi) rapid Wang [22] (same algorithm for defining and identifying budding, same microscope field features; quantification done in two steps: first, binary categorization of each HPF (classified as positive if ≥1 buds *vs.* negative if 0 buds); next, binary categorization of the whole case (classified as high if ≥50% of all HPFs examined are positive *vs.* low if <50% are positive)) (reviewed in [2,13,34]).

The reproducibility of tumor budding assessment varies across different studies. Most authors have evaluated reproducibility using Cohen’s κ coefficient (no agreement, κ < 0; slight, κ = 0–0.20; fair, κ = 0.21–0.40; moderate, κ = 0.41–0.60; substantial, κ = 0.61–0.80; almost perfect, κ = 0.81–1). Intra-observer agreement ranges from substantial [20] to almost perfect [16,29], while inter-observer agreement ranges from fair [13,34] to moderate [22,24,34] to substantial [25,26,27,28,35,36] to almost perfect [14]. It is not clear whether pan-cytokeratin immunostaining or H&E staining yields better reproducibility, as the two large comparative studies published to date reported conflicting results ([13,31]; also reviewed in [2]). Assessment of tumor budding by general pathologists with no special interest in this phenomenon, as opposed to experienced gastrointestinal pathologists, does not appear to alter reproducibility [28], which might prove useful for wide implementation of the method. Moreover, inter-observer reproducibility appears to improve with training [22,35].

A study performed on pT1-pT4 CRC samples attempted to compare one categorical and four quantitative methods, both under H&E staining and pan-cytokeratin immunostaining: (i) Hase *et al.*, 1993, categorical [5]; (ii) Nakamura *et al.*, 2005, categorical (reviewed in [2,13,34]); (iii) Ueno *et al.*, 2002, quantitative [16]; (iv) Ueno *et al.*, 2004, quantitative (reviewed in [2,13,34]); (v) conventional Wang, quantitative [22], adapted [13]; (vi) rapid Wang, quantitative [22], adapted [13]. The quantitative methods proposed by Ueno *et al.*, 2002 [16], and Wang *et al.*, 2009 [22], proved to be the most effective in both the H&E staining and pan-cytokeratin immunostaining settings, while the subjective method proposed by Hase *et al.*, 1993 [5] was the least effective. The inter-observer reproducibility was fair for all methods under both H&E staining and pan-cytokeratin immunostaining [13].

A more recent comparative study, conducted on stage II (pT3-4N0) CRC, asessed two categorical and five quantitative methods under pan-cytokeratin immunostaining (CK22, which recognizes a cocktail of cytokeratins ranging from 40–68 kDa): (i) Hase *et al.*, 1993, categorical [5]; (ii) Nakamura *et al.*, 2005, categorical (reviewed in [2,13,34]); (iii) Ueno *et al.*, 2004, quantitative (reviewed in [2,13,34]); (iv) conventional Wang *et al.*, 2009, quantitative [22], adapted (quantification by counting all the buds, without averaging; classification as low (≤9 buds) *vs.* high (>9 buds) as this cutoff value best stratified patients with respect to survival); (v) rapid Wang *et al.*, 2009 [22], quantitative, adapted (HPF classified as negative (≤9 buds) *vs.* positive (>9 buds); classification as high (≥50% of the HPFs analyzed per patient are positive) *vs.* low (<50% of the HPFs analyzed per patient are positive)); (vi) 1 HPF method, proposed by the authors of the study, quantitative (assessment by bud counting; region of interest spanning microscope field displaying maximal budding, followed by selection of 1 HPF as seen at 400×, field area = 0.49 mm^2^); and (vii) 10 HPF method, proposed by the authors of the study [36], quantitative (assessment by bud counting; region of interest spanning microscope field displaying maximal budding, followed by selection of 10 HPF as seen at 200×, field area = 0.49 mm^2^; quantification by counting all the buds, without averaging). All methods, except for (iv) conventional Wang, adapted and (v) rapid Wang, adapted, found correlations between budding and infiltrating growth pattern. No method found any correlation between budding and gender, tumor location, histopathological type, grade, venous invasion, or lymphatic invasion. Interestingly, the 1 HPF and 10 HPF methods were the only ones to reveal an independent correlation between budding and cancer-specific survival (CSS) time and also showed comparably high discriminatory power across the entire follow-up period, with the 10 HPF having a slight edge for 5 and 10 years postoperatively. The 1 HPF and 10 HPF methods showed excellent inter-observer reproducibility (intraclass correlation coefficient (ICC) = 0.83 and 0.91, respectively). The inter-observer reproducibility was moderate to substantial for conventional Wang, adapted (Cohen's κ= 0.46–0.62), moderate for rapid Wang, adapted (κ = 0.46–0.58), moderate for Nakamura *et al.*, 2005 (κ = 0.41–0.52), fair to moderate for Ueno *et al.*, 2004 (κ = 0.39–0.56) and fair to moderate for Hase *et al.*, 1993 (κ = 0.29–0.51) [34]. While the 10 HPF and 1 HPF methods appear to be the only ones revealing the prognostic significance of budding, the authors recommend using the 10 HPF over the 1 HPF method, as it provides superior inter-observer reproducibility due to increased sampling of the region of interest, which increases statistical significance and, therefore, consistency of the findings [34]. However, the best cutoff value for stratifying patients under these two methods remains an open question (also see below).

A recent review encompassing studies done using H&E staining, studies done using pan-cytokeratin immunostaining, and studies comparing the two methods, respectively (all these studies having assessed budding in either CRC, colon cancer, or rectal cancer (RC)), concluded that quantitative methods using pan-cytokeratin immunostaining improved detection as compared to quantitative methods based on H&E staining. However, the prognostic value and the inter-observer reproducibility remained the same, irrespective of the staining method used [2]. This conclusion should, nevertheless, be interpreted with caution for two reasons. First, it was not derived from a meta-analysis, so there is no quantification of the statistical significance of any of the claims from the reviewed studies. As the authors themselves acknowledge, a meta-analysis would have been impossible anyway because of the great methodological heterogeneity across the tumor budding literature [2]. Therefore, the review could only look for very general claims, such as the independent correlation between budding and survival. Second, even though pan-cytokeratin immunostaining does not appear to have more prognostic value than the H&E staining, this should probably be rephrased. Namely, it might be, rather, that tumor budding is such a strong prognostic factor that the statistical correlation remains even when budding is underestimated under less accurate detection methods. The very fact that it consistently turned out to be prognostic, despite the methodological heterogeneity used, strongly supports this idea. The ultimate purpose would be to use budding score as a tool for selecting a subset of patients with a poorer prognosis than is indicated by the other histopathological parameters, and to reassign them to the proper therapeutic regimen. While not as important for establishing the prognostic significance of budding in a patient set, the slight edge that pan-cytokeratin immunostaining has over H&E staining might, thus, become paramount for assigning particular patients to appropriate prognostic and therapeutic categories.

A question as yet incompletely addressed is how frequent tumor budding really is among CRC patients. By comparing the results of various studies, some authors have even tried to extract a rough prevalence, as seen either by H&E staining or pan-cytokeratin immunostaining [2]. However, the heterogeneity of the studies under review, not only in terms of methodology used, but also in terms of patient characteristics, renders these attempts unable to yield a true quantitative picture. Rather, those percentages should be interpreted as a general warning that tumor budding is frequently encountered. However, in the same vein, it can be seen that, for the subset of stage I CRC patients (reviewed in [3]), the methodology of Ueno *et al.*, 2004 (see [2,3,13,34] and the references therein) appears to yield fairly consistent percentages among different studies. While the heterogeneity of the studies make it hard to observe similar trends for the other methodologies, this indirectly gives further validation to the 1 HPF method [34], which is an improved version of the Ueno *et al.*, 2004 method, with increased inter-observer reproducibility. This, in turn, gives further credit to the 10 HPF method [34], which, as shown above, appears to perform even better than the 1 HPF method.

Last, but not least, there is still a great deal of debate around choosing the cutoff value for stratifying patients, *i.e.*, the intensity of tumor budding, which best separates them into prognostic categories, followed by appropriate therapeutic decisions. Many reports tended to favor the cutoff = 10 buds [16,19,20,25,32,36], with some reports having relied on either the Kaplan-Meier plots themselves [20] or receiver operating characteristic curves with survival as the endpoint, which were constructed based on a subset of the patients and run for calibration [36]. Other studies, however, chose this value arbitrarily or based on previous publications [16,19,25]. Another two studies, one relying on Kaplan-Meier plots [18] and the other one running a receiver operating characteristic curve for one budding assessment methodology under study [34], found the close cutoff value = 9 buds. Some recent publications suggested using budding score as a continuous variable rather than introducing cutoff values ([31,34]; also reviewed in [3]).

We next discuss the impact of budding on CRC outcome and the utility of its addition to the standard pathological diagnostic practice in CRC patients.

## 3. Tumor Budding in Colorectal Cancer: Clinical Significance

In the absence of a consensus methodology, it is difficult to discuss the real prognostic significance of tumor budding. Several attempts have been made to review the various studies published to date (see, for example, the excellent reviews by De Smedt and colleagues, van Wyk and colleagues, and Koelzer and colleagues [1,2,3]). The numerous studies have, not only used highly diverse methodologies for defining and assessing the phenomenon, but they have also relied on equally diverse patient inclusion criteria. The various patient populations studied range from one single stage (or even sub-stage), or type, of CRC, to all stages and types combined.

### 3.1. Tumor Budding Association with Recurrence and Survival

In CRC, high budding correlated with overall recurrence [18,19,21,37], both locoregional [38] and distant [29,38]. Among distant recurrence sites, the most frequently involved were the peritoneal cavity [37,38,39] and liver [38,39]. Consequently, high budding also correlated with lower 5-year disease-free survival (DFS) ([17,18,21,25], although see [40]). In metastatic CRC patients treated with cetuximab or panitumumab, high budding correlated with lower progression-free survival ([24]). In CRC, high budding correlated with lower 5-year overall survival (OS) ([5,17,18,19,37,39], although see [15]), 10-year OS [5,39], 5-year CSS [22,25,28,36,38,39], and 10-year CSS [32]. The correlation with lower OS is maintained even for metastatic CRC [41].

Particular attention has been paid to stage II CRC [22,25,36,38,39,42] (extensively reviewed in [1,2,3]), due to the uncertainty regarding proper risk stratification of these patients (for a thorough discussion see, for example, [43]). A recently conducted meta-analysis reported that in stage II CRC patients, particularly among pT3N0M0 cases, high budding correlated with increased risk of death at five years. Interestingly, the hazard-ratio for death did not differ across pathological methodologies for tumor budding assessment (as proposed in [16] *vs.* [5] *vs.* other authors) or tumor site (colon *vs.* colorectal) [42]. However, a potential (and probably unavoidable) flaw of this conclusion is that the cutoff value for ‘high-grade budding’ varied among the studies included in the meta-analysis. Budding appears to be an important additional prognostic factor that can correctly identify patients who are candidates for adjuvant therapy (reviewed in [3]).

In colon cancer, high budding correlated with lower 5-year recurrence-free survival (RFS), 5-year OS [5] (although see [23]), 10-year OS [5], and 5-year CSS [27]. In RC, high budding correlated with recurrence, both local and distant (most notably liver metastases) [14] and consequently, with lower 5-year DFS [20]. High budding also correlated with lower 5-year OS [5,16,37] and 10-year OS [5].

### 3.2. Tumor Budding Association with Other Factors Traditionally Linked to Poor Prognosis

Apart from recurrence and survival, tumor budding also correlates with various prognostic features (reviewed in [1,3]). In CRC patients, high budding correlated with higher overall stage [28,36], higher T stage [18,21,32,36] (but see [26]), higher N stage [17,18], higher Dukes stage [5,18], tumor dedifferentiation [5], infiltrating growth pattern [22,26,36], lymphatic invasion [5,17,18,21,26], venous invasion [5,17,18,19,21,26,28,38], lymphovascular invasion [22,25,36], perineural invasion [5,17], nodal metastasis [15,19,21,32,36,37,40], and distant metastasis [37] (but see [26]). Budding intensity did not correlate with tumor grade [17,26,28] (although see [36,40]). In metastatic CRC, high budding predicted non-response to anti-epidermal growth factor receptor (EGFR) agents [24].

Several studies assessed tumor budding separately in each of the two anatomic categories of CRC (*i.e.*, colon cancer and RC). In colon cancer patients, high budding correlated with high tumor grade [23], infiltrating growth pattern [27], venous invasion [23], lymphovascular invasion [27], and infiltration of a free serosal surface [23]. In RC patients, high budding correlated with higher tumor grade [16], extramural spread [16], lymphatic invasion [6,14], venous invasion [6], extramural venous invasion (*i.e.*, to vessels outside the muscularis propria) [16], nodal metastasis [6,14,16], and lymphocyte infiltrate [16]. Apart from the association with high tumor grade, the results are consistent with, and bring further support to, the findings in CRC.

## 4. Tumor Budding in Colorectal Cancer: Implementation in Clinical Practice

It has been suggested that tumor budding assessment might help identify patients at risk for worse outcome in the following contexts: (i) patients with pT1 lesions or malignant polyps endoscopically resected with increased risk for nodal metastasis; (ii) patients with stage II disease who might benefit from adjuvant therapy; and (iii) patients with RC undergoing biopsy who might benefit from neoadjuvant therapy (reviewed in [31]).

Although both staining methods are equally reproducible (see above), pan-cytokeratin immunostaining should probably be used instead of H&E staining whenever feasible [31] (also see above). Comparisons of the two staining methods revealed that H&E staining tended to underestimate tumor budding [13,21,29,31,44], while pan-cytokeratin immunostaining frequently helped visualize numerous buds intermingled with stromal fibroblasts [29], yielding three- to four-fold higher tumor bud counts than H&E staining [31]. Moreover, one recent study reported that, among relatively inexperienced pathologists (<5 years of practice), pan-cytokeratin immunostaining markedly increased inter-observer reproducibility as compared to H&E staining. The increase was weaker among the average-experienced pathologists (5–10 years of practice) and weakest among the most experienced pathologists (>10 years of practice). This, however, suggests that pan-cytokeratin immunostaining might be helpful for inexperienced pathologists [44]. Differently put, pan-cytokeratin immunostaining should probably be used instead of H&E staining because budding assessment, if implemented into clinical practice, will be performed by pathologists with various levels of experience (see also [28] for another discussion regarding budding assessment in relation to experience).

An impartial selection from among the numerous identification and quantification methods should be attempted. Quantification across 10 HPF should probably be used for optimal viewing of surgical samples, while the more restrictive 1 HPF method might be reserved for small samples, such as biopsies or pT1 stage tumors [31]. While these two methods performed very well in a seemingly well-conducted study [34], it should be noted, however, that the other older, established methods used in the study did not yield any prognostic correlation. Since these latter methods also served as “controls”, against which the novel 10 HPF and 1 HPF methods could be validated, the fact that they did not see any correlation with outcome (contrary to previous publications, see [2,3,13,34] and the references therein) is somewhat surprising. It might, therefore, be necessary to test the 10 HPF and 1 HPF protocols against other patient sets to confirm their power.

A tentative unifying algorithm for tumor budding assessment has been proposed: (i) tissue block selection: seek for block displaying highest budding or infiltrative growth pattern (frequently associated with budding, can be used as surrogate selection criterion); (ii) budding definition: clusters of ≤5 cells in the peritumoral stroma, with clearly visible nuclei; (iii) budding identification: cytokeratin immunostaining, exclude cytoplasmic pseudofragments, fragmented glands, mucinous pools, and necrosis; and (iv) budding quantification: for surgical samples, use 10 HPF method (scan at low power, 40×–100× for ten areas displaying maximal budding; count at high power, 400×, 0.49 mm^2^; either use cutoff value, e.g., ≥10 per HPF (as proposed in [36]) or total bud count across the 10 HPF, which can be plotted against continuous hazard ratio-to-bud count graphs, as previously described [31,34]; for biopsy samples and malignant polyps (scarce material for pathologic assessment), use 1 HPF method (same magnification and field area as for the 10 HPF method) (see [3] and the references therein).

## 5. Tumor Budding in Other Cancer Types

The consistency among the published reports noting the prognostic significance of tumor budding in CRC has stimulated research to determine if this is possible in other cancer types. Most of these analyses were undertaken during the last decade. Among the most comprehensively studied cancer sites are the esophagus [45,46,47,48,49,50,51,52], pancreas [53,54,55,56], head and neck [57,58,59], lung [60,61,62], and breast [63,64]. These emerging sub-fields relied largely on concepts and methodologies already developed in colorectal cancer studies, sometimes with minor adaptations, such as the definition of tumor buds proposed by Ueno and colleagues [16], which has apparently become the accepted norm [45,46,47,48,49,50,51,54,56,57,58,60,61,62,63,64,65,66,67,68]. Consequently, the heterogeneity of tumor budding research, at least in terms of methodology, is decreasing, which brings the field one step closer to being introduced in clinical guidelines.

*Esophageal cancer.* Most studies have examined tumor budding in squamous cell carcinoma of the esophagus (reviewed in [69]), where high budding correlated with larger tumor size [45,46,49], higher T stage [45,46,49,51], higher overall stage [45,46,51], infiltrative invasion pattern [50], deeper invasion [50], lymphatic invasion [46,49,50,51], vascular invasion [46,49,50], lymphovascular invasion [45], perineural invasion [45], nodal metastasis [46,49,50], intramural metastases [46], and incomplete excision of the tumor [45]. High budding correlated with lower 5-year DFS [49], lower 3-year OS [45,46,50] and 5-year OS [46,47,49,51]. In a recent meta-analysis, tumor budding correlated with lower OS [59]. In metastatic squamous cell carcinoma, high tumor budding was correlated with an observed non-response to neoadjuvant chemotherapy [47]. The significance of budding in adenocarcinoma of the esophagus (reviewed in [69]) is less clear. Budding correlated with non-intestinal phenotypes (Lauren classification) and non-tubular or non-papillary phenotypes (WHO classification), higher tumor grade, higher pT stage, lymphatic invasion, venous invasion, perineural invasion [70], nodal metastasis [70,71], and recurrence [71], and high budding correlated with an almost 20-fold increase in risk for recurrence [71]. However, neither peri-tumoral nor intra-tumoral budding correlated independently with OS [70]. Despite this weaker prognostic correlation for adenocarcinoma, one study, which included both patients with squamous cell carcinoma and patients with adenocarcinoma, found that high budding correlated with higher grade, higher T stage, higher overall stage, nodal metastasis, lower inflammatory response, incomplete excision of the tumor, and lower OS [48].

*Stomach cancer.* Higher budding correlated with lower 3-year OS and 5-year OS. The correlation remained significant for the patient subset with intestinal type tumors [72].

*Ampullary cancer.* High budding correlated with non-intestinal histology, which is more aggressive, infiltrating growth pattern, higher tumor grade, higher T stage, lymphatic invasion, perineural invasion, and nodal metastasis [73]. High budding also correlated with lower 3-year OS and 5-year OS [73].

*Gallbladder cancer.* Budding correlated with higher tumor grade at the invasive front, higher T stage, and nodal metastasis [65].

*Pancreatic ductal adenocarcinoma (PDAC).* For unknown reasons, studies have used very different ways to interpret tumor budding, ranging from solitary cell infiltration [53], to the average between peri- and intra-tumoral bud counts [54], to the maximal bud count among peri- and intra-tumoral budding, combined [55,56]. High budding correlated with higher tumor grade [53,55], pT stage [56], lymphatic invasion [53,56], lymphovascular invasion [55], perineural invasion [55], and nodal metastasis [53] (marginal correlation in [55]). High budding also correlated with lower DFS [56], OS [53,54,56], and CSS [55].

*Head and neck cancer.* Tumor budding is a strong predictor of poor prognosis in head and neck cancer (reviewed in [59]). In oral squamous cell carcinoma, high budding correlated with lower DFS [67]. In tongue squamous cell carcinoma, high budding correlated with higher tumor size, tumor grade, overall clinical stage, and nodal metastasis [58]. High budding also correlated with lower 5-year OS [58] and lower CSS (reviewed in [59]). In nasopharyngeal carcinoma, high budding correlated with higher T stage, higher overall clinical stage, lymphatic invasion, vascular invasion, and nodal metastases. Furthermore, high budding correlated with lower OS ([74]; also reviewed in [59]). In laryngeal carcinoma, high budding correlated with lower metastatic DFS [57]. In squamous cell carcinoma of the external auditory canal, high budding correlated with lower CSS [68].

*Lung cancer.* In lung adenocarcinoma, budding correlated with higher overall pathologic stage [60], infiltrating growth pattern [61], schirrhous (*i.e.*, rich in dense connective tissue) stromal type [61], lymphatic invasion [60,61], vascular invasion [60], pleural invasion [60], and nodal metastases [60,61]. Budding correlated with lower OS [61], including lower 5-year OS [60]. In lung squamous cell carcinoma, budding correlated with tumor size, higher overall pathologic stage, lymphatic invasion, venous invasion, pleural invasion, and nodal metastases [62]. Budding also correlated with lower 5-year OS and 5-year CSS [62].

*Invasive ductal breast cancer.* In invasive ductal breast cancer, high budding correlated with larger tumor size [63], lymphatic invasion [75], lymphovascular invasion [63] and nodal metastasis [75] (also see [63]). High budding also correlated with lower 5-year OS [63] and shorter CSS [64]. The correlation with lower 5-year OS remained significant for the patient subsets with good prognosis (*i.e.*, small tumor size, no lymphovascular invasion, and no lymph node metastases, respectively) [63]. Similarly, the correlation with lower CSS remained significant for the patient subset without nodal metastases [64].

*Endometrial cancer.* In endometrial cancer, high budding correlated with advanced overall stage (III + IV) and deep invasion of the myometrium (≥50%) [66]. High budding also correlated with lower 5-year OS [66].

*Normal-appearing epithelium adjacent to primary carcinomatous lesions.* Although insufficiently studied, it has been reported that epithelia in the vicinity of CRC primary tumors also display budding. Such detached cell clusters have been observed to infiltrate neighboring lymphatics, which made some suggest that CRC metastasis might also occur from a tumor cell niche located in the surrounding, seemingly normal epithelium [76]. Similar findings have been reported (albeit by the same group) in breast and prostate cancer (reviewed in [76]). However, according to the definitions published to date, tumor budding refers to the presence of malignant (as opposed to normally looking epithelial) cells in the tumor stroma. Therefore, these observations should be interpreted with caution, not only due to their absolute novelty and replication by one single group of authors, but also in terms of definition, which does not fit into the malignant cell-centered definition of tumor budding.

It should, however, not be forgotten that the only cancer type where budding is already recognized by the UICC and authoritative guidelines as a prognostic factor is the CRC. In addition, CRC is the type of cancer where the methodologies for assessing tumor budding have been reviewed the most. Thus, for CRC, the distance towards reaching a consensus and implementing tumor budding assessment into clinical practice is the shortest. While the studies conducted in other cancer types do indeed depict a similar trend in terms of prognostic significance, the relative methodological heterogeneity applied to a comparatively lower number of patients analyzed per cancer type make it more difficult to assess the real importance of tumor budding in these patients.

## 6. Intra-Tumoral Budding

Although tumor budding most often has been described as occurring at the invasive front of the tumor, this is rather an approximation. Some authors noticed that similar structures are also present (albeit less frequently) in other areas of the malignant tissue, and decided to count them as true buds (see, for example, [6,22] for CRC, [54,55,56] for pancreatic cancer, and [75] for breast cancer). Several questions might arise. First, does intra-tumoral budding have prognostic significance? Second, is that significance similar to peri-tumoral budding? Third, when might intra-tumoral budding be preferable for use as a histologic marker instead of peri-tumoral budding?

Intra-tumoral budding essentially means the presence of buds within the main tumor mass. Several authors have studied this phenomenon separately [26,30,32,75,77], most notably in CRC. Intra-tumoral budding has generally been defined using the same criteria as for peri-tumoral budding (see above). The cutoff value chosen for defining intra-tumoral budding has been either <5 cells, as in the earlier criteria proposed by Ueno and colleagues (see above) [26] or as clusters of ≤5 cells, according to the criteria proposed more recently by Karamitopoulou and colleagues (see above) [30,75,77]. As for assessment, the various authors used either (i) the semiquantitative method of Nakamura (see above) [26]; (ii) the quantitative 1 HPF method of Karamitopoulou (see above), either unchanged [30] or adapted with cutoff value for stratification = 6 buds [77]; or (iii) the quantitative 10 HPF method of Karamitopoulou (see above), adapted with cutoff value for stratification = 10 buds [75]. In CRC, high intra-tumoral budding correlated with higher tumor grade, higher T stage, lymphatic invasion, vascular invasion, and nodal metastasis. Moreover, intra-tumoral budding was independently associated with a shorter survival time [77]. This is roughly similar to the prognostic significance of peri-tumoral budding; in fact, in the surgical samples analyzed, intra-tumoral budding correlated with peritumoral budding [77]. In breast cancer, intra-tumoral budding yielded conflicting statistical results between observers [75].

Intra-tumoral budding might become a particularly attractive histopathologic marker in preoperative biopsies, which usually cannot properly assess the tumor invasive margin [26] and, consequently, peri-tumoral budding. In fact, in CRC patients, high intra-tumoral budding in biopsy samples correlated with high peri-tumoral budding in surgical samples from the same patients [26]. In biopsy samples, high intra-tumoral budding correlated with higher tumor grade [26], advanced pT stage [30] (although see [26]), venous invasion [30] (although see [26]), lymphatic invasion [30] (although see [26]), lymph node metastasis [26,30] and distant metastasis [26,30] at the time of diagnosis, but not with tumor growth pattern or perineural invasion [26]. In RC patients, high intra-tumoral budding in pre-treatment biopsy samples predicted non-response to neoadjuvant therapy and cancer-specific death [78].

## 7. Epithelial-Mesenchymal Transition (EMT) and Partial EMT

Epithelial-mesenchymal transition is a multistep dynamic cellular phenomenon in which epithelial cells lose their cell–cell adhesion and gain migratory and invasive traits that are typical of mesenchymal cells [79]. EMT and its reverse process, mesenchymal-epithelial transition (MET), are physiologic processes that play key roles during embryonic development, wound healing, and tissue repair. Aberrant activation of EMT is considered to be a hallmark of cancer metastasis (reviewed in [79,80,81]).

EMT involves loss of membranous localization of the epithelial marker E-cadherin and an increase in levels of one or more mesenchymal markers, such as vimentin, N-cadherin, α-smooth muscle actin, or fibronectin, along with a loss of polarized function of epithelial cells. The E-cadherin usually sequesters β-catenin at cell-cell contacts; a decrease in its membranous levels can result in nuclear translocation of β-catenin and consequent activation of Wnt/β-catenin signaling as EMT progresses [82]. An increase in the levels of one or more of the EMT-inducing transcription factors, such as ZEB1, ZEB2, SNAI1, SNAI2 (SLUG), TWIST, GSC, and FOXC2 also often accompanies EMT progression (reviewed in [83]).

Recent findings suggest that EMT and MET are not all-or-none responses, *i.e.*, switching between purely epithelial and purely mesenchymal phenotypes [81], but rather multi-state processes, ranging from purely epithelial to purely mesenchymal via one or several intermediate phenotypes. These intermediate phenotypes, the number of which is still a matter of debate, have been variously and sometimes interchangeably referred to as ‘intermediate EMT’ [84,85], ‘intermediate mesenchymal’ [86], ‘incomplete EMT’ [87,88,89], ‘semi-mesenchymal’ [90], ‘hybrid epithelial/mesenchymal’ [91,92,93,94,95], ‘EMT-like’ [96,97,98], ‘partial EMT’ [99,100,101,102,103], or ‘metastable’ [104,105] phenotype(s). For the purpose of this review, the intermediate phenotype(s) will be referred to as “partial EMT”.

Most of the studies reporting partial EMT have been conducted at a cell population level (as opposed to a single-cell level), thereby raising questions of whether partial EMT represents (i) a mixed cell population composed of epithelial and mesenchymal cells; (ii) a pure cell population composed of hybrid epithelial/mesenchymal cells (E/M); or (iii) a mixed cell population composed of epithelial, mesenchymal, and hybrid epithelial/mesenchymal cells. Several recent theoretical studies of EMT regulatory networks have predicted that a hybrid E/M phenotype can exist at a single-cell level [85,92,93,95,102]. Concomitantly, several experimental studies using fluorescence activated cell sorting in a few cell lines have identified a subpopulation of cells that can co-express epithelial and mesenchymal markers [85,106], and a recent report showed that lung cancer cells can maintain a hybrid E/M phenotype at a single-cell level stably over multiple passages [107]. Cells in a hybrid E/M phenotype are expected to display both adhesion (an epithelial trait) and migration (a mesenchymal trait), hence exhibiting collective cell migration. Therefore, the cells moving collectively during wound healing [108], mammary morphogenesis [109], and cancer, as clusters of circulating tumor cells [110], are likely manifestations of the hybrid E/M phenotype, and a similar proposition can be made for tumor buds encompassing more than one cell.

The magnitude of change in the levels of the epithelial and mesenchymal markers can be used to distinguish between EMT and partial EMT phenotype. While EMT often associates with an almost complete loss of membranous localization of E-cadherin, *i.e.*, a ‘cadherin switch’ from E-cadherin to N-cadherin, single-cell co-expression of an epithelial marker (usually E-cadherin) and a mesenchymal marker can be considered as the most reliable set to identify a hybrid E/M phenotype. For example, (i) Andriani *et al.* [111] investigated single-cell co-expression of SNAI2 and E-cadherin to identify the relative abundance of different subpopulations (epithelial, hybrid E/M, and mesenchymal) in lung cancer cell lines (A549, LT73, and H460); (ii) Grosse-Wilde *et al.* [106] sorted the breast cancer cell line HMLER based on an epithelial surface marker CD24 and a mesenchymal stem cell marker CD44, and showed that CD24+/CD44+ cells co-expressed epithelial and mesenchymal genes equivalently; (iii) Lu and colleagues [92] predicted via mathematical modeling that the single-cell co-expression of ZEB and E-cadherin levels can account for a hybrid E/M phenotype; (iv) Jolly and colleagues [107] showed that H1975 lung cancer cells co-express E-cadherin and vimentin stably over two months in culture; and (v) Jeevan and colleagues [112] indicated that metastatic brain tumor samples can co-express E-cadherin and vimentin at a single-cell level. Furthermore, relative levels of E-cadherin and vimentin at a population level has also been used to categorize panels of cell lines into epithelial, partial EMT, and mesenchymal [86,94,113]. Notably, as already discussed earlier, classification of a population as partial EMT does not necessarily imply a stable single-cell hybrid E/M phenotype.

## 8. Tumor Budding and Partial EMT

Tumor budding often is observed at the invasive edge due to detachment of a few tumor cells from the main tumor mass (reviewed in [1,2,3,114]). Consistently, the membranous localization of E-cadherin, the key cell-cell adhesion molecule in epithelial cells, is relatively low in tumor buds of CRC [10], esophageal cancer [51], PDAC [53,54], head and neck cancer [58,59,67], lung cancer [60], and invasive ductal breast cancer [63]. Loss of membranous localization of E-cadherin is a hallmark of EMT [33], thus tumor buds have been proposed to be “EMT-like” (see [63] and the references therein). However, do they really mirror EMT, or a partial EMT?

Evidence connecting tumor budding and EMT (*i.e.*, purely mesenchymal phenotype) is scarce. One of the first studies to address budding in CRC found truly single cancer cells ahead of the invasive front, both on 2D sections and serial sections); the single cells extended cytoplasmic projections similar to lamellipodia. However, sectioning artifacts could not be ruled out, which rendered the finding of single cells equivocal [8]. More convincing piece of evidence comes from esophageal squamous cell cancer, where high budding correlated with EMT [51]. Similarly, in PDAC, budding correlated with both reduced E-cadherin and increased vimentin levels, which suggests that it might be the morphological feature of EMT [53].

By contrast, most evidence seems to connect tumor budding with partial EMT. In PDAC, the membranous localization of E-cadherin and beta-catenin in tumor buds was lower (at a cell population level) as compared to the tumor center. However, vimentin levels in tumor buds did not show any correlation (either direct or inverse) with E-cadherin levels [54]. In addition, few tumors showed vimentin positivity, which suggests that most EMT occurrences in PDAC patients are partial EMT [54]. In invasive ductal breast cancer, budding cell populations had lower membranous localization of E-cadherin and higher vimentin cytoplasmic levels than center tumor cells [63]. In tongue squamous cell carcinoma, the membranous localization of E-cadherin was reduced in tumor buds as compared to the main tumor mass, while vimentin levels were positive in tumor buds but not in the main tumor mass. High budding correlated with reduced membranous localization of E-cadherin and positive vimentin levels [58]. Furthermore, in a study conducted on various tumor types (CRC, liver metastases of CRC, PDAC, lung adenocarcinoma, and invasive breast ductal cancer), Bronsert and colleagues found no evidence of single-cell migration. While not irrefutably dismissing their existence, this observation still suggests that single-cell migration is very rare (0.000%–0.003% of total tumor cell number) [10], and, therefore, that cancer cell invasion relies mostly, if not entirely, on collective cell migration, rather than single cell migration, which in turn suggests that some epithelial cell–cell adhesions persist. Hence, these findings imply that not all the cells become purely mesenchymal, which is indicative of a partial EMT.

The expression and/or localization of other EMT-related markers further support the correlation between tumor budding and partial EMT. In lung cancer, tumor buds display decreased levels of membranous β-catenin [62], while in CRC and oral squamous cell carcinomas they display reduced levels of miR-200 family [115,116] which are strong inhibitors of ZEB1/2 [117,118,119]. Furthermore, buds exhibit increased levels of laminin-5γ2 [60,68] (also see [59] and references therein), an extracellular matrix glycoprotein that is expressed at the leading edge [120] of collectively migrating cells during wound healing [80], a manifestation of partial EMT [108]. Therefore, the decreased levels of epithelial markers argue for tumor budding to be correlated with EMT. However, association of tumor buds with increased levels of mesenchymal markers remains inconclusive. Although tumor buds of breast carcinoma display higher levels of vimentin [63], and oral squamous cell carcinoma tumor buds have significantly upregulated fibronectin [116], only a fraction of tumor samples of oral squamous cell carcinoma underwent a full ‘cadherin switch’ from E-cadherin to N-cadherin [67]. Moreover, lung cancer buds do not express ZEB1 (the predicted master regulator of EMT [92,121,122]) at a significantly upregulated level as compared to the main tumor mass [62]. In PDAC buds, ZEB1 is significantly higher, but increased nuclear localization of β-catenin is absent [123]. RNA-sequencing analysis of tumor buds in oral squamous cell carcinomas showed that the protein level of ZEB1 was higher as compared to that in the tumor center, but lower than that in adjacent stromal cells [116]. This observed trimodal distribution of ZEB corroborates with Lu and colleagues suggesting that (miR-200/ZEB) double negative feedback loop enables the three phenotypes—*i.e.*, epithelial (high miR-200, low ZEB), mesenchymal (low miR-200, high ZEB), and hybrid E/M (medium miR-200, medium ZEB) [92]. Collectively, these studies suggest that tumor buds do not have a full-blown stable mesenchymal phenotype; instead they are manifestations of a partial EMT. Importantly, they also call for a more quantitative approach to characterize the levels of many tumor budding markers.

In this setting of tumor budding as a histological expression of partial EMT, several studies have suggested that tumor budding might mirror a real single-cell hybrid E/M phenotype. In esophageal squamous cell cancer, tumors displaying high budding showed weak membranous E-cadherin and strong cytoplasmic vimentin immunostaining, while tumors exhibiting low budding showed strong membranous localization of E-cadherin and no cytoplasmic vimentin [51]. In PDAC, high budding correlated with reduced E-cadherin and increased vimentin levels [53]. Interestingly, E-cadherin, although reduced, was still at least weakly positive in most of these cells [53], which supports the idea of a hybrid E/M phenotype. In oral squamous cell carcinoma, tumor buds showed loss of membranous localization of E-cadherin and increased cytoplasmic levels of vimentin, at both cell population and single-cell level [67]. Moreover, in the study conducted on various tumor type by Bronsert and colleagues, the budding tumor cells displayed loss of cell polarity (*i.e.*, a morphological shift towards rounded and spindle-like phenotype), decreased levels of E-cadherin, decreased membrane localization coupled to increased cytoplasmic localization of E-cadherin, and increased nuclear ZEB1 immunostaining at a single-cell level [10]. However, because the hallmarks of EMT (spindle-like shape, E-cadherin loss and ZEB1 levels) were rarely seen even among tumor buds, and because of the absence of single cell migration, their findings might rather be interpreted as a collective migration with partial EMT [10] showing a hybrid E/M phenotype, instead of a full-blown mesenchymal phenotype.

To further decode any causal connections between EMT and tumor budding, molecular events that trigger tumor budding should be characterized beyond clinical studies. Various EMT-inducing signaling pathways, such as TGF-β and Wnt [83], have also been reported to be active in tumor budding [116,124]. Future *in vivo* experimental studies that investigate how various molecular players govern EMT [125] at multiple stages of the invasion-metastasis cascade should explore how tumor budding at both primary and metastatic tumor sites is affected.

## 9. Proliferation/Quiescence and Cancer Stem Cells in Tumor Buds: Further Connection with EMT

In CRC, tumor buds showed lower nuclear Ki-67 levels (*i.e.*, lower proliferation) and higher nuclear p16^INK4^, cyclin D1, and β-catenin levels [126,127,128], as compared to the main tumor mass [126,127]. Since the aforementioned markers correlated, not only at a cell population level, but also at a single cell level as seen from serial sections, as opposed to the main tumor mass which displayed inverse levels [127], it has been suggested that the p16^INK4^ increase sequesters Cdk4 in the cytosol, thus enabling nuclear cyclin D1 to bind Cdk2, which competes with (and prevents) binding of cyclins A and E to Cdk2, resulting in the decreased proliferation reported in tumor buds [128]. Most cells in the tumor buds are in this low or non-proliferative state [129]. Similarly, in breast cancer, budding cells showed lower Ki-67 levels as compared to the main tumor mass [63]. Interestingly, in lung cancer, tumor buds exhibited decreased nuclear levels of geminin, a key cell cycle regulator as compared to the main tumor mass [62], which might offer an alternative explanation for the co-occurrence of decreased E-cadherin and β-catenin levels with a non-proliferative state in budding cells (see [62] and the references therein). Moreover, in CRC, tumor buds show lower levels of the apoptosis markers caspase-3 and M30 [130] and the proliferative marker Ki-67 [131]. Collectively, these findings suggest that tumor buds are quiescent, which further supports a putative involvement of EMT in tumor budding because (i) it corroborates with the ‘go-or-grow,’ or proliferation/migration dichotomy, hypothesis ([132], recently demonstrated in a developmental context by Matus and colleagues [133] who showed that the invasive phenotype requires cell cycle arrest); (ii) induction of EMT has been shown to be capable of both blocking the cell cycle and conferring resistance to apoptosis in multiple contexts [134,135,136,137]; and (iii) overexpression of miR-200 and consequent MET can restore sensitivity to apoptosis and anoikis, a form of cell-death triggered by separation from the extracellular matrix [138]. However, these observations do not necessarily resolve whether tumor budding is a surrogate for either a partial EMT or a complete EMT.

Tumor buds express higher levels of stem-cell surface markers such as CD133 (reviewed in [139]) and aldehyde dehydrogenase 1 [74], indicating a cancer stem-cell like trait associated with tumor budding. Interestingly, budding tumor cells expressing aldehyde dehydrogenase 1 were directly involved in aggressiveness and lower OS [74]. Recent theoretical [140,141] and experimental studies [106,142,143] investigating the interplay between EMT and stemness have indicated that cells in a partial EMT phenotype can be more stem-like than both purely epithelial or purely mesenchymal cells, thereby bolstering the proposed association of tumor budding and partial EMT. Therefore, tumor buds at the invasive edge can be considered to be the realization of the proposed ‘migrating cancer stem cells’ [144].

## 10. Conclusions

**Figure 1:**
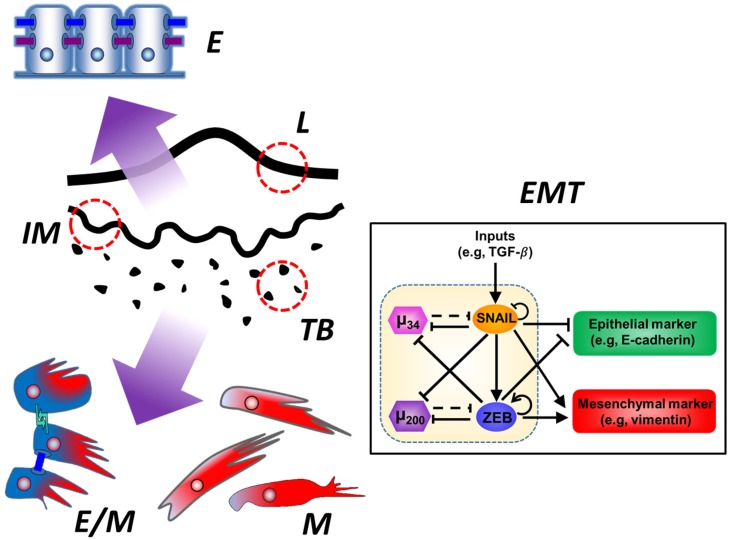
Tumor budding as a partial epithelial-mesenchymal transition. *(Left)* Tumor budding (TB) occurs mainly at the invasive tumoral margin (IM) which is opposite to the luminal margin (L). Studies have shown that tumor buds as a whole show a loss of epithelial markers and a gain of mesenchymal markers as compared to the main tumor mass. These features are compatible with epithelial-mesenchymal transition, which is why budding is considered to be the histological expression of the same. However, not all the cells in a tumor bud are purely mesenchymal, so budding rather mirrors a partial epithelial-mesenchymal transition. While the main tumor mass is most notably composed of purely epithelial (E) cells, the tumor buds are greatly enriched in purely mesenchymal (M) and even hybrid epithelial-mesenchymal (E/M) cells. *(Inset)* Epithelial-mesenchymal transition (EMT) is controlled by a core regulatory circuit composed of four players: two microRNAs, known as miR-34 (μ_34_, pink hexagon) and miR-200 (μ_200_, purple hexagon), respectively, and two transcription factors, referred to as SNAIL (orange ellipse) and ZEB1/2, which stands for Zinc finger E-box-binding homeobox-1 or -2 (ZEB, blue ellipse), respectively. The microRNAs promote the epithelial phenotype, characterized by synthesis of epithelial markers such as E-cadherin (green rounded rectangle). The transcription factors promote the mesenchymal phenotype, characterized by the synthesis of mesenchymal markers such as vimentin (red rounded rectangle). The core EMT regulatory circuit consists of two mutually inhibitory feedback loops, miR-34/SNAIL and miR-200/ZEB, respectively. Solid arrows depict transcriptional activation, with circled arrows showing self-activation. Solid bar-headed arrows depict transcriptional inhibition, with circled bar-headed arrows showing self-inhibition. Dotted bar-headed arrows represent microRNA-mediated regulation at a translational level. The relative concentrations of the four players at any given time point alter the resultant state of the core EMT regulatory circuit, thus altering the expression of epithelial and mesenchymal genes and consequently the synthesis of epithelial and mesenchymal markers. By this mechanism, the core EMT regulatory circuit governs the cellular phenotype, which can exist in (or dynamically switch between) a purely epithelial (E), a hybrid epithelial/mesenchymal (E/M), and a purely mesenchymal (M) phenotypic state. See text for more details on the relationship between EMT at a molecular scale and tumor budding at a histologic scale.

Tumor budding is a phenomenon encountered in various cancers whereby the primary tumor sends numerous fingerlike projections, or buds, towards the neighboring stroma, some of which eventually detach from the main tumor mass as small cell clusters. It is widely believed that tumor buds provide the histological basis for invasion and metastasis. Tumor budding correlates with poor outcomes across all the cancer types in which it has been described. The down-regulation of epithelial markers and concomitant up-regulation of mesenchymal markers, which have been reported in tumor buds, have raised the idea that tumor budding is the morphological expression of EMT (Figure 1). Most EMT processes in tumor buds, however, are not complete, which rather suggests that tumor buds undergo partial EMT, with at least a subset of tumors displaying a true hybrid, single-cell E/M phenotype in their buds.

## Abbreviations

CRC	colorectal cancer
CSS	cancer-specific survival
DFS	disease-free survival
E/M	epithelial/mesenchymal
EMT	epithelial-mesenchymal transition
H&E	hematoxylin and eosin
PDAC	pancreatic ductal adenocarcinoma
RFS	recurrence-free survival
OS	overall survival
RC	rectal cancer
